# Morphological features of the nasal cavities of hawksbill, olive ridley, and black sea turtles: Comparative studies with green, loggerhead and leatherback sea turtles

**DOI:** 10.1371/journal.pone.0250873

**Published:** 2021-04-29

**Authors:** Chiyo Kitayama, Keiichi Ueda, Mariko Omata, Taketeru Tomita, Shingo Fukada, Shogo Murakami, Yoshiaki Tanaka, Akihiro Kaji, Satomi Kondo, Hiroyuki Suganuma, Yuki Aiko, Atsuru Fujimoto, Yusuke K. Kawai, Masashi Yanagawa, Daisuke Kondoh

**Affiliations:** 1 Everlasting Nature of Asia (ELNA), Ogasawara Marine Center, Ogasawara, Tokyo, Japan; 2 Okinawa Churaumi Aquarium, Motobu, Okinawa, Japan; 3 Okinawa Churashima Research Center, Motobu, Okinawa, Japan; 4 Shimane Aquarium, Hamada, Shimane, Japan; 5 Everlasting Nature of Asia (ELNA), Yokohama, Kanagawa, Japan; 6 Obihiro University of Agriculture and Veterinary Medicine, Obihiro, Hokkaido, Japan; Institute of Marine Research, NORWAY

## Abstract

We analyzed the internal structure of the nasal cavities of hawksbill, olive ridley and black sea turtles from computed tomography images. The nasal cavities of all three species consisted of a vestibule, nasopharyngeal duct and cavum nasi proprium that included anterodorsal, posterodorsal and anteroventral diverticula, and a small posteroventral salience formed by a fossa of the wall. These findings were similar to those of green and loggerhead sea turtles (Cheloniidae), but differed from those of leatherback sea turtles (Dermochelyidae). Compared to the Cheloniidae species, the nasal cavity in leatherback sea turtles was relatively shorter, wider and larger in volume. Those structural features of the nasal cavity of leatherback sea turtles might help to suppress heat dissipation and reduce water pressure within the nasal cavity in cold and deep waters.

## Introduction

Sea turtles are ocean-living Testudines. Most sea turtles are now endangered, and thus understanding their ecology and sensory organs, including the nose, is more imperative than ever. There are seven sea turtle species; green (*Chelonia mydas*), loggerhead (*Caretta caretta*), hawksbill (*Eretmochelys imbricata*), olive ridley (*Lepidochelys olivacea*), Kemp’s ridley (*L*. *kempii*), flatback (*Natator depressus*), and leatherback (*Dermochelys coriacea*) [1]. Due to a distinctive appearance, some green turtles are often referred to as black sea turtles (*C*. *mydas agassizii*). While these morphological differences have led to the suggestion that black sea turtles are a distinct subspecies [2], categorization as such is still under discussion, and the Turtle Taxonomy Working Group [1] concluded that genetic analyses [3] do not indicate that black sea turtles are a separate subspecies. This study was conducted from the standpoint that black turtles are a subspecies of green turtles, because we focus on their morphology.

**Fig 1:**
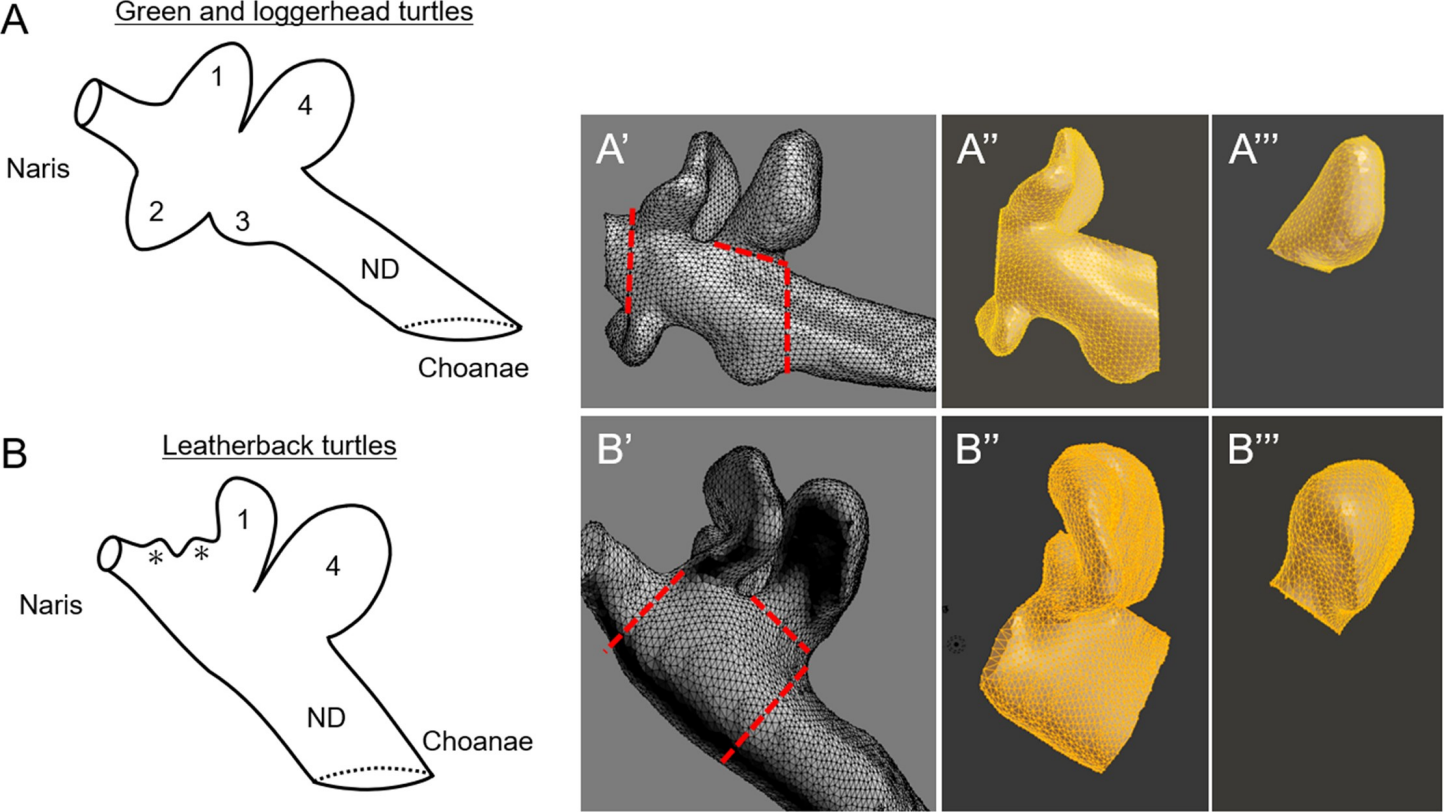
Drawing schema of internal nasal structure of sea turtles and extraction for volume measurement in this study. (A and B) Green and loggerhead (A) and leatherback (B) sea turtles. Left lateral view. All species have anterodorsal (1) and posterodorsal (4) diverticula, and nasopharyngeal duct (ND). Leatherback sea turtles lack anteroventral (2) diverticulum and posteroventral fossa (3), but two additional fossae (*) are anteriorly to anterodorsal diverticula (1). (A’ and B’) Extraction of intermediate and olfactory regions from nasal cavity of Cheloniidae species (A’) and dermochelyid leatherback sea turtle (B’). Red dashed lines, section cuts. (A” and B”) Intermediate region. (A”‘ and B”‘) Olfactory region.

Odor detection in vertebrates plays important roles in their ecology, such as breeding, feeding and detecting predators [4,5]. Sea turtles can detect volatile and water-soluble odorants with high sensitivity [6–9]. We previously described the morphological and histological features of the nasal cavities of green sea turtles [10,11]. The nasal cavity comprises the vestibule, cavum nasi proprium, and nasopharyngeal duct [12]. The cavum nasi proprium is mainly lined by sensory epithelia and has four sections, comprising anterodorsal, anteroventral and posterodorsal diverticula and a posteroventral small salience formed by a fossa on the wall (Fig 1A). The cavum nasi proprium of turtles in general has an upper area where water hardly enters and a lower area where water easily enters, and Parsons (1959) defined the former as “olfactory region” and the latter as “intermediate region” [12]. In green sea turtles, water easily enters the anterodorsal and anteroventral diverticula and the posteroventral fossa of the wall, but hardly enters the posterodorsal diverticulum [10], indicating that the posterodorsal diverticulum of sea turtles corresponds to the olfactory region whereas the other structures in the cavum nasi proprium correspond to the intermediate region.

Although the nasal cavities are similar between loggerhead and green sea turtles, the architectural features of the nasal cavity of leatherback sea turtles significantly differ [13]. The cavum nasi proprium of leatherback sea turtles has two dorsal diverticula and two small saliences formed by fossae on the wall in the dorsal side, anterior to the diverticula, without obvious structures in the ventral side [13] (Fig 1B). The morphological features of the nasal cavity vary by species even among sea turtles; thus the nasal cavity in other sea turtle species should be clarified. Here, we visualized the internal structures of the nasal cavity of hawksbill, olive ridley and black sea turtles using computed tomography (CT). We also compared some index values, such as the volume ratio of olfactory region to the whole cavum nasi proprium to determine how the noses of various species differ from each other.

## Materials and methods

### Animals

We analyzed CT-data acquired from two hawksbill, *Eretmochelys imbricata* (ID: H-1 and H-2), three olive ridley, *Lepidochelys olivacea* (ID: O-1, O-2 and O-3) and three black, *Chelonia mydas agassizii* (ID: B-1, B-2 and B-3) sea turtles. Table 1 shows the straight carapace length and width, body weight, and source of each individual. Images of all animals except O-3 were acquired by CT without anesthesia, and with careful consideration of their welfare during regular physical check-ups prior to the present study between 2016–2020 at Okinawa Churashima Foundation (OCF), Japan. The head of a dead turtle (O-3) was assessed by CT at Obihiro University of Agriculture and Veterinary Medicine (OUAVM) under the approval of the Animal Care and Use Committee at OUAVM (Notification number 28–44).

**Table 1:** Features of analyzed sea turtles.

Species	ID	Sex	Age (y)	Status	Straight carapace length (cm)	Straight carapace width (cm)	Body weight (kg)	Source
Hawksbill	H-1	Female	Unknown	Alive	79.0	59.0	59.2	OCF
	H-2	Unknown	7	Alive	Unknown	Unknown	18.0	OCF
Olive ridley	O-1	Female	Unknown	Alive	52.6	49.8	18.2	OCF
	O-2	Male	Unknown	Alive	56.0	50.4	32.6	OCF
	O-3	Unknown	Unknown	Dead, Frozen	51.8	41.8	19.8	SAA
Black	B-1	Female	Unknown	Alive	82.0	66.0	91.8	OCF
	B-2	Unknown	2	Alive	38.7	31.1	8.1	OCF
	B-3	Unknown	2	Alive	38.3	30.8	7.2	OCF

OCF, Okinawa Churashima Foundation; SAA, Shimane AQUAS Aquarium.

### Computed tomography

Images were acquired using a SOMATOM Definition AS+ (Siemens Healthcare Japan, Tokyo, Japan) under the following conditions: 120–140 kV, 70–287 mA, and 0.6 mm slice thickness, and an Aquilion TSX-201A (Toshiba Medical Systems Corporation, Otawara, Japan) at OUAVM as described [13]. Data stored in DICOM format (approximately 200 projections for each head) were processed for reconstruction into three-dimensional (3D) images using AZE Virtual Place software (AZE Ltd., Tokyo, Japan). The internal architecture of the nasal cavity was visualized in the software mode for outline detection of the lungs.

### Length and volume measurements

We measured the head length, the distance from the naris to the choanae, and the diameter of central region of the nasopharyngeal duct, of all specimens studied here, in addition to seven (two green, two loggerhead and three leatherback) sea turtles by using CT-data obtained in previous studies [10,13]. The 3D images of the nasal cavities have been revealed in these seven individuals [10,13], but any measurements have not been taken yet. The DICOM files were processed using RadiAnt DICOM Viewer software (Medixant, Poznan, Poland) in Angio software mode to measure the head length, and in Airways mode to measure the length and diameter of the nasal cavity.

Structural details of the cavum nasi proprium were clearly determined in all specimens studied here and three (two green and one leatherback) sea turtles in previous studies [10,13], and volumes of the intermediate and olfactory regions of these turtles were measured. The DICOM files were processed using Fiji/ImageJ software (https://imagej.nih.gov/ij/). The internal structure of the nasal region was extracted from sequenced 8-bit inverted images on a threshold range of 200–255, reconstructed into 3D-images with a 3D-Viewer plugin (display as surface; threshold, 100; resampling factor, 2) and imported as STL files. Three-dimensional images of the left side of the nasal cavity were divided into intermediate and olfactory regions (Fig 1) using Blender software (www.blender.org), then the volume of each region was measured using Volume Statistics in the 3D-Print Toolbox add-on of Blender software.

## Results

**Fig 2:**
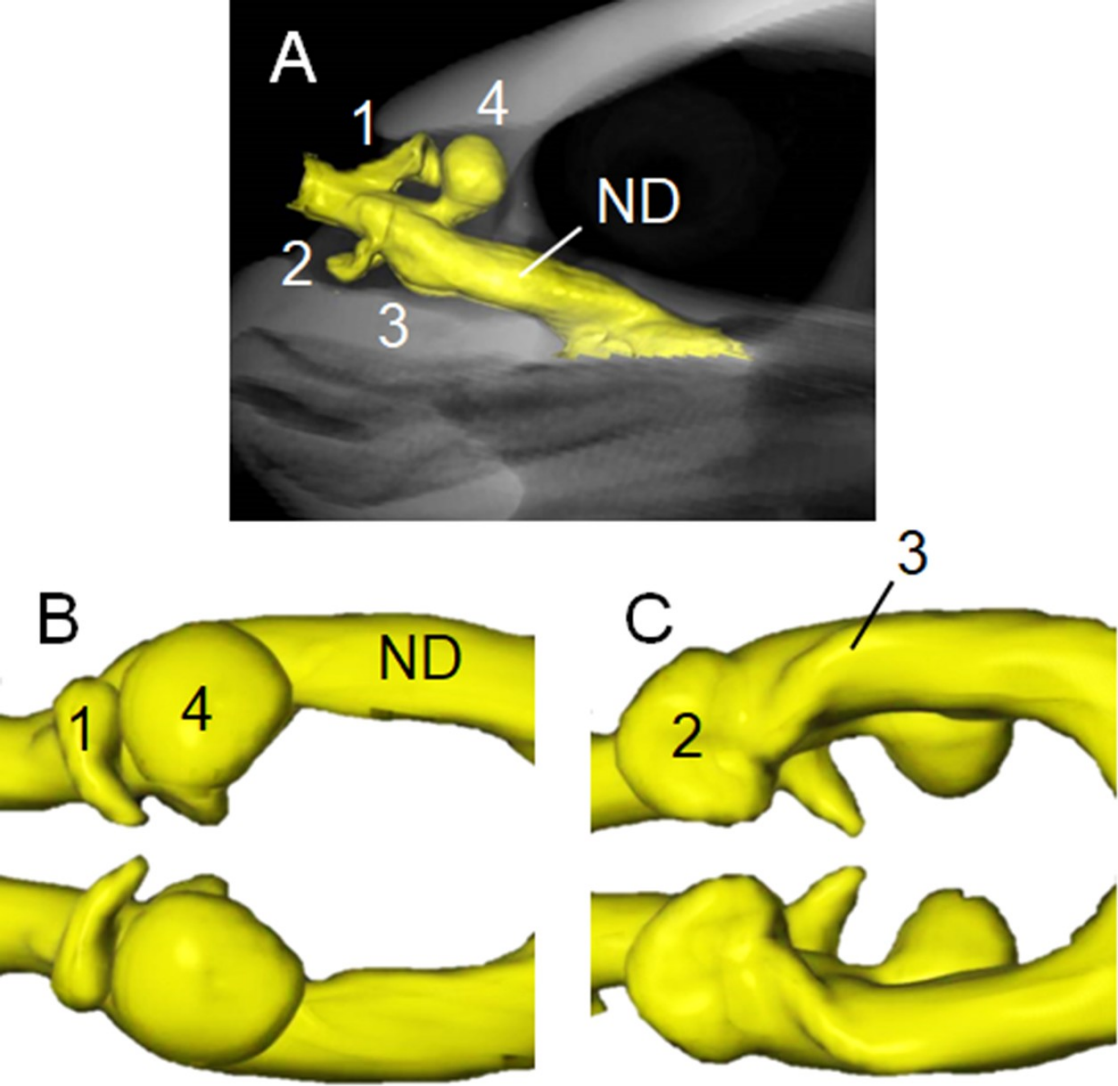
Three-dimensional reconstructions based on computed tomography images of nasal cavity of hawksbill sea turtle. (A) Left lateral view of internal nasal cavity (yellow) at anterior region of skull. Dorsal (B) and ventral (C) views of nasal cavity. Anterodorsal (1), posterodorsal (4) and anteroventral (2) diverticula, and small posteroventral salience formed by fossa on wall (3) in cavum nasi proprium anterior to nasopharyngeal duct (ND). See also S1 Video.

**Fig 3:**
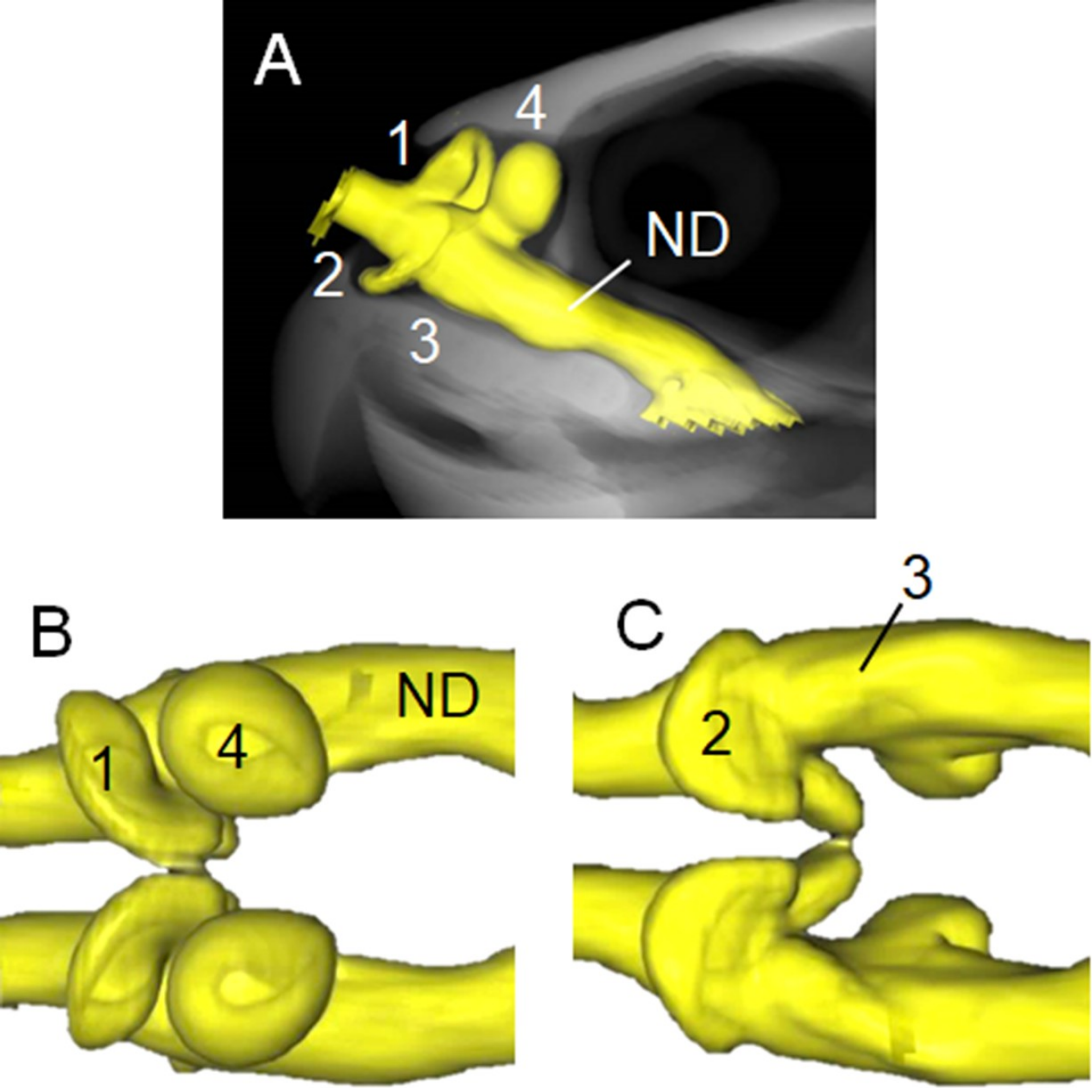
Three-dimensional reconstructions based on computed tomography images of nasal cavity of olive ridley sea turtle. (A) Left lateral view of nasal cavity structure (yellow) at anterior region of skull. Dorsal (B) and ventral (C) views of nasal cavity. Anterodorsal (1), posterodorsal (4) and anteroventral (2) diverticula, and small posteroventral salience formed by fossa on wall (3) in cavum nasi proprium anterior to nasopharyngeal duct (ND). See also S2 Video.

**Fig 4:**
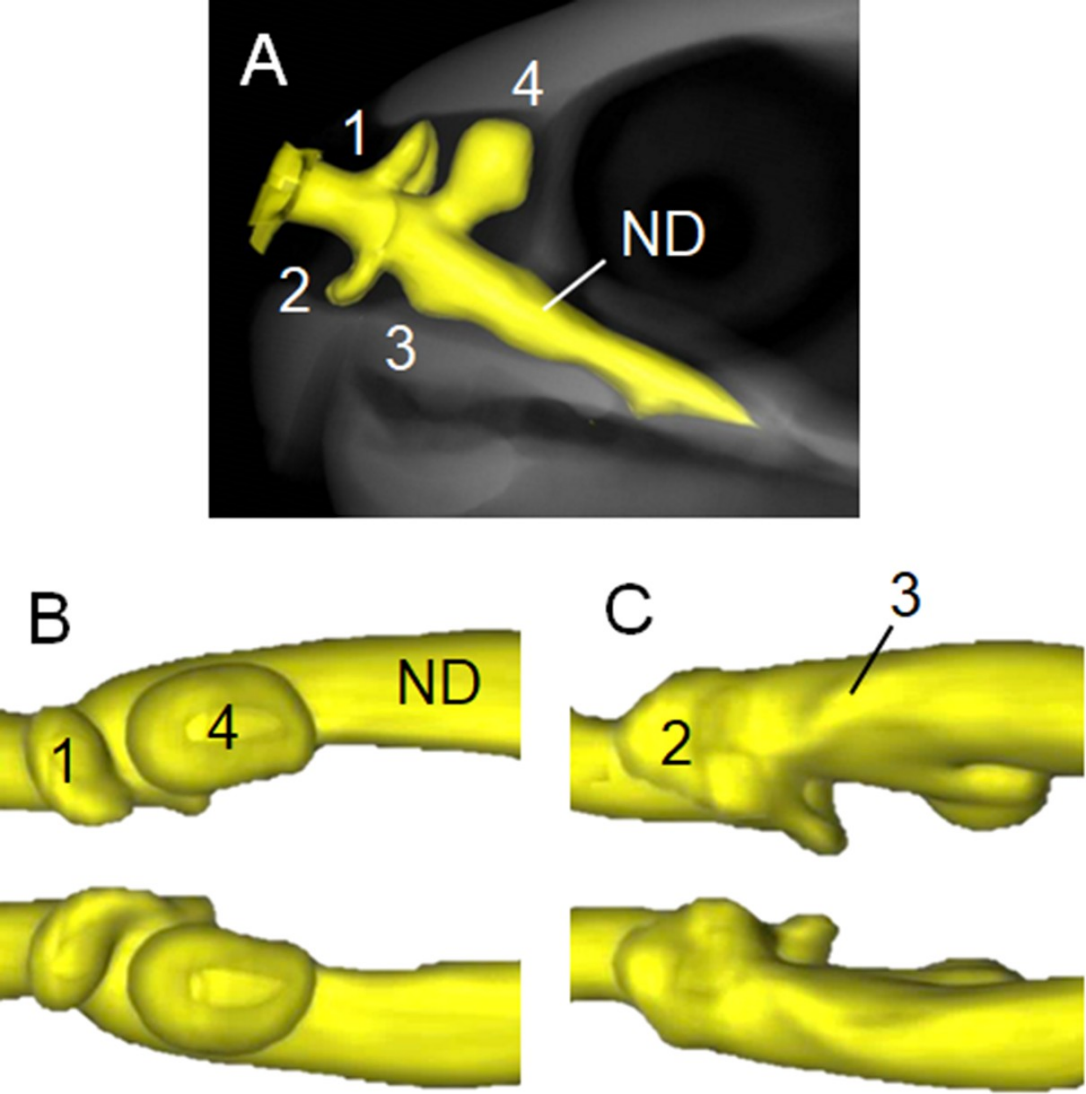
Three-dimensional reconstructions based on computed tomography images of nasal cavity of black sea turtle. (A) Left lateral view of nasal cavity structure (yellow) at anterior region of skull. Dorsal (B) and ventral (C) views of nasal cavity. Anterodorsal (1), posterodorsal (4) and anteroventral (2) diverticula, and small posteroventral salience formed by fossa on wall (3) in cavum nasi proprium anterior to nasopharyngeal duct (ND).

Figs 2–4 respectively show the internal structures of the nasal cavities of hawksbill, olive ridley and black sea turtles. The nasal cavities of all three species are a pair of tubes from the nostril to the choana opening into the buccal cavity. The cavum nasi proprium consists of significant anterodorsal, posterodorsal and anteroventral diverticula, and a small posteroventral salience formed by a fossa on the wall. A vestibule is found as a short tubular region from the nostril to the cavum nasi proprium, and a nasopharyngeal duct is a long tubular structure from the cavum nasi proprium to the choana.

The structure of the posterodorsal diverticulum (Figs 2–4; b) is that of a balloon, whereas those of the anterodorsal (Figs 2–4; a) and anteroventral (Figs 2–4; c) diverticula resemble broad beans. The posteroventral salience formed by a fossa on the wall (Figs 2–4; d) is located posterior to the anteroventral diverticulum and runs from the anteromedial to posterolateral sides, but it is not prominent compared with the other sections. The structure of the nasal cavities in all examined animals is bilaterally symmetrical.

Table 2 lists the measurements taken from total 15 sea turtles measured here. Ratios of the nasal cavity length and width to the head length are within a specific range for all Cheloniidae species (0.29–0.37 and 0.030–0.053, respectively), but those in leatherback sea turtles are out of that range (0.20–0.24 and 0.058–0.062, respectively) (Table 3). On the other hand, the volume ratio of the olfactory region in the whole cavum nasi proprium do not differ among species (range, 0.24–0.36) (Table 3), although the total volume of the cavum nasi proprium divided by head length is much larger in the leatherback sea turtle (0.246) than other species (range, 0.036–0.135) (Table 3).

**Table 2:** Measurements of left half of nasal structure.

Species	ID	Head length (cm)	Distance from naris to choanae (cm)	Diameter of center of nasopharyngeal duct (cm)	Volume of intermediate region (cm^3^)	Volume of olfactory region (cm^3^)
Hawksbill	H-1	16.8	5.0	0.71	0.93	0.44
	H-2	11.8	3.8	0.57	0.46	0.16
Olive ridley	O-1	12.8	4.3	0.62	0.71	0.38
	O-2	13.4	4.9	0.59	0.73	0.28
	O-3	13.1	4.9	0.69	1.34	0.43
Black	B-1	16.4	5.7	0.70	1.14	0.65
	B-2	9.2	2.9	0.47	0.29	0.11
	B-3	9.3	2.9	0.41	0.28	0.11
Green	Cm-1^†^	13.8	4.1	0.41	0.34	0.15
	Cm-2^‡^	18.0	5.8	0.66	0.93	0.37
Loggerhead	Cc-1^‡^	20.3	6.7	0.83	ND	ND
	Cc-2^‡^	20.8	6.1	0.67	ND	ND
Leatherback	Dc-1^‡^	18.6	4.5	1.07	ND	ND
	Dc-2^‡^	21.3	4.3	1.33	3.78	1.47
	Dc-3^‡^	20.1	4.2	1.18	ND	ND

ND, Not determined.

^†^An individual reported in [10].

^‡^Individuals reported in [13].

**Table 3:** Indicators of the nasal morphology analyzed in this study.

Species	ID	Distance from naris to choanae: head length	Diameter of nasopharyngeal duct: head length	Volume of cavum nasi proprium: head length	Volume of olfactory region: volume of cavum nasi proprium
Hawksbill	H-1	0.30	0.042	0.082	0.32
	H-2	0.32	0.048	0.053	0.26
Olive ridley	O-1	0.34	0.048	0.085	0.35
	O-2	0.37	0.044	0.075	0.28
	O-3	0.37	0.053	0.135	0.24
Black	B-1	0.35	0.043	0.109	0.36
	B-2	0.32	0.051	0.043	0.28
	B-3	0.31	0.044	0.042	0.28
Green	Cm-1^†^	0.30	0.030	0.036	0.31
	Cm-2^‡^	0.32	0.037	0.072	0.29
Loggerhead	Cc-1^‡^	0.33	0.041	ND	ND
	Cc-2^‡^	0.29	0.032	ND	ND
Leatherback	Dc-1^‡^	0.24	0.058	ND	ND
	Dc-2^‡^	0.20	0.062	0.246	0.28
	Dc-3^‡^	0.21	0.059	ND	ND

ND, Not determined.

^†^An individual reported in [10].

^‡^Individuals reported in [13].

## Discussion

We found that the morphological features of the nasal cavities in hawksbill (*E*. *imbricata*), olive ridley (*L*. *olivacea*) and black (*C*. *mydas agassizii*) sea turtles were similar to those of other Cheloniidae, namely the green (*C*. *mydas*) and loggerhead (*C*. *caretta*) sea turtles [10,13]. The cavum nasi proprium of all three of these species comprised anterodorsal, posterodorsal and anteroventral diverticula, and a small posteroventral salience formed by a fossa on the wall, and the relative size of the olfactory region to the cavum nasi proprium was also similar among sea turtle species. Water easily enters the anterodorsal and anteroventral diverticula and the posteroventral fossa on the wall, but hardly enters the posterodorsal diverticulum in the nasal cavity of green sea turtles [10]. In addition, the anterodorsal—anteroventral diverticula, the posteroventral fossa, and the posterodorsal diverticulum within the nasal cavity of green sea turtles are each covered by different types of sensory epithelium [10]. Therefore, the anterodorsal and anteroventral diverticula and the posteroventral fossa appear to receive water-soluble odorants, whereas the posterodorsal diverticulum likely detects volatile odorants [14]. The nasal cavities of hawksbill, olive ridley and black sea turtles are structurally similar; thus the distribution of water and air is likely to be similar in the nasal cavities between these species and green sea turtles. On the other hand, the architectural features of the nasal cavity of the dermochelyid leatherback sea turtle (*D*. *coriacea*) (Fig 1; S3 Video) differ considerably from those of Cheloniidae [13]. Although leatherback sea turtles also have two diverticula and two fossae on the wall, they are all located on the dorsal side of the nasal cavity [13]. In addition, volume of the cavum nasi proprium in leatherback sea turtles is much larger than that in Cheloniidae species. In the future, histological analyses of the nasal cavity are required to guess the olfactory function in leatherback sea turtles.

The airway in the nasal cavity of the leatherback sea turtles is shorter in the anteroposterior direction and wider than those of other sea turtles. The secondary palate is well-developed in Cheloniidae species [15], whereas it is absent in leatherback sea turtles [16]. Thus, the short nasopharyngeal duct in leatherback turtles seems to reflect the absence of a secondary palate. The leatherback is the largest of the sea turtle species, and its unique features clearly differ from those of the Cheloniidae. For example, Cheloniidae species are generally distributed in tropical, subtropical and temperate waters, whereas adult leatherback sea turtles are prevalent in both tropical and near-freezing waters [17], and they can dive to depths of > 1,000 m [18,19]. Some morphological characteristics of this Dermochelyidae species, apart from their nasal cavities, such as the relatively soft and flexible shell [20] and compressible elliptical cartilaginous tracheal tube [21] suitable for deep diving, indicate a close relationship to its ecology. We speculate that the architectural differences in the nasal cavities of sea turtle species also reflect its ecological distinctiveness. For example, the short and wide airway, in addition to large cavum nasi proprium, of leatherback sea turtles might help to suppress heat dissipation and reduce water pressure within the nasal cavity in cold and deep waters.

## Conclusions

The noses of hawksbill, olive ridley and black sea turtles have anterodorsal, posterodorsal and anteroventral diverticula, and a posteroventral fossa. These findings are similar to those of green and loggerhead sea turtles (Cheloniidae) [10,13], but different from that of the leatherback sea turtle (Dermochelyidae) [13]. Since those groups are also diverse ecologically, we suggest the structural variety of the nasal cavity might be related to this ecological and evolutionary diversity of sea turtles.

## Supporting information

S1 Video 3D reconstruction model of nasal cavity of hawksbill sea turtle. (M4V)

S2 Video 3D reconstruction model of nasal cavity of olive ridley sea turtle. (M4V)

S3 Video 3D reconstruction model of nasal cavity of leatherback sea turtle. (M4V)
